# Prise en charge de la hernie diaphragmatique congénitale en Afrique sub-Saharienne: l’expérience du Centre Hospitalier National d’Enfants Albert Royer au Sénégal

**DOI:** 10.11604/pamj.2022.41.185.30907

**Published:** 2022-03-08

**Authors:** Papa Alassane Mbaye, Doudou Gueye, Mbaye Fall, Florent Tshibwid A Zeng, Cheikh Seye, Ndeye Fatou Seck, Lissoune Cissé, Ndeye Aby Ndoye, Aloïse Sagna, Gabriel Ngom

**Affiliations:** 1 Service de Chirurgie Pédiatrique, Hôpital d’Enfants Alber Royer, Université Cheikh Anta Diop, Dakar, Sénégal,; 2 Service de Chirurgie Pédiatrique, Hôpital Aristide Le Dantec, Université Cheikh Anta Diop, Dakar, Sénégal,; 3 Laboratoire d´Anatomie, Université Alioune Diop, Bambey, Sénégal

**Keywords:** Hernie diaphragmatique congénitale, prise en charge, Afrique, Sénégal, Albert Royer, Congenital diaphragmatic hernia, management, Africa, Senegal, Albert Royer

## Abstract

**Introduction:**

la hernie diaphragmatique congénitale est une pathologie rarement rapportée en Afrique et dont la présentation peut être précoce ou tardive. Le pronostic dépend essentiellement des malformations associées. Le but de cette étude est de rapporter notre expérience au Centre Hospitalier National d´Enfants Albert Royer de Dakar au Sénégal.

**Méthodes:**

il s´agit d´une étude rétrospective considérant les patients pris en charge pour hernie diaphragmatique congénitale entre Janvier 2010 et Décembre 2019.

**Résultats:**

douze patients ont été inclus, dont l´âge moyen était de 8,9 mois. Les hernies de Bochdalek ont été retrouvées chez 10 patients. Les symptômes les plus fréquents étaient respiratoires (83,3%), puis digestifs (41,6%). La radiographie thoraco-abdominale a permis d´évoquer le diagnostic chez tous nos patients. Trois patients ont bénéficié d´une stabilisation préopératoire. Tous les patients ont bénéficié d´une laparotomie, le sac herniaire était présent chez 10 patients et 50% des patients avaient un défect mesurant entre 5 et 10 cm. Les suites opératoires ont été simples chez 10 patients et un décès a été enregistré, concernant un patient polymalformé.

**Conclusion:**

la hernie diaphragmatique congénitale est une réalité dans notre milieu, se présentant le plus souvent au-delà de la période néonatale. Son pronostic est globalement bon dans notre contexte.

## Abstract (en)

**Introduction:**

congenital diaphragmatic hernia has been rarely reported in Africa. It can manifests early or late. Prognosis mainly depends on associated malformations. The purpose of this study is to report our experience in the Albert Royer National Children's Hospital, Dakar, Senegal.

**Methods:**

we conducted a retrospective study of patients treated for congenital diaphragmatic hernia between January 2010 and December 2019.

**Results:**

twelve patients were enrolled, with an average age of 8.9 months. Bochdalek hernias were detected in 10 patients. The most common symptoms were respiratory symptoms (83.3%), followed by digestive symptoms (41.6%). Thoraco-abdominal X-ray was used to make a diagnosis in all patients. Three patients underwent preoperative stabilization. All patients underwent laparotomy. Hernia sac was found in 10 patients, and 50% of patients had a defect measuring between 5 and 10 cm. The postoperative course was simple in 10 patients; a polymalformed patient died.

**Conclusion:**

congenital diaphragmatic hernia is a reality in our environment; it most often manifests beyond the neonatal period. Prognosis is generally good in our context.

## Introduction

La hernie diaphragmatique congénitale (HDC) est une pathologie rare, se caractérisant par un défect diaphragmatique unilatéral ou bilatéral, de taille variable [1,2]. Grâce au développement de l´échographie anténatale, diagnostic anténatal est largement possible dans les pays à haut revenu et presque inexistant dans les pays à faible revenu où la découverte peut être précoce ou tardive [3-6]. Contrairement à la découverte néonatale où la détresse respiratoire met directement en jeu le pronostic vital, dans la découverte tardive, les symptômes respiratoires et digestifs sont généralement modérés, pouvant être aigus ou chroniques [7]. Le diagnostic est généralement retenu après une radiographie thoraco-abdominale et le traitement chirurgical est actuellement relégué au second plan après la stabilisation des patients présentant une hypertension artérielle pulmonaire et/ou une hypoxie [1,4]. La survie dépend des malformations associées [3].

La HDC est un sujet relativement peu étudié en Afrique sub-Saharienne, cela justifie notre revue rétrospective dont le but est de rapporter les aspects épidémiologiques, mais aussi diagnostiques et thérapeutiques des HDC au service de chirurgie pédiatrique du Centre Hospitalier National d´Enfants Albert Royer (CHNEAR) de Dakar.

## Méthodes

**Conception de l´étude:** il s´agit ici d´une étude rétrospective descriptive transversale. Nous avons recruté les patients pris en charge dans notre service entre janvier 2010 et décembre 2019. La collecte des données s´est faite de manière rétrospective, entre janvier et mars 2020.

**Cadre:** elle a été menée au sein du CHNEAR de Dakar, au Sénégal. C´est un service fondé en 2010 qui, avec les services de l´Hôpital d´Enfants de Diamniadio, de l´Hôpital Aristide Le Dantec et de Kaolack constituent les seuls services publics de chirurgie pédiatrique au Sénégal.

**Participants:** les critères d´inclusion étaient: a) des patients âgés de moins de 16 ans; b) les diagnostics confirmés dans notre service; c) durant la période allant du premier janvier 2010 au 31 décembre 2019. Les critères d´exclusion étaient: a) les hernies diaphragmatiques traumatiques; b) les dossiers médicaux sans renseignements suffisants malgré les tentatives de les compléter; c) les hernies diaphragmatiques congénitales avec décès préopératoire et; d) les hernies paraœsophagiennes. Les patients ont été sélectionnés après avoir satisfait aux critères d´inclusion.

**Variables:** les paramètres recueillis ont inclus l´âge, le sexe, l´âge maternel, la réalisation de l´échographie anténatale, le lieu, le terme et la voie d´accouchement, l´apgar et le poids de naissance, la voie de recrutement, le motif de référence. Les renseignements diagnostics ont été: les malformations associées, les symptômes, les examens complémentaires. Les données thérapeutiques ont inclus: l´utilisation de la stabilisation, la voie d´abord, la présence d´un sac, la taille du défect diaphragmatique, les organes herniés, la résection du sac lors de la réparation, le type de suture, le type de fil et la mise en place ou pas d´un drain thoracique. Les indicateurs des suites opératoires ont inclus le délai de reprise du transit, la durée d´hospitalisation et les complications postopératoires.

**Source:** le recueil a été fait à partir des dossiers médicaux et des registres des comptes rendus opératoires.

**Collecte et analyse des données:** la collecte des données a été effectuée à partir d´une fiche d´enquête. Pour réduire le risque des biais d´information et d´enregistrement, les données ont été recueillis par deux personnes et les données finales ont résulté de la fusion des listes initiales.

**Taille de la population d´étude:** la taille de l´échantillon a été déterminée par le nombre de patients ayant satisfait aux critères d´inclusion sans avoir d´élément des critères d´exclusion.

**Analyses statistiques:** les analyses avaient pour objectif de déterminer les moyennes des données quantitatives (âge au diagnostic) et les fréquences des données qualitatives (fréquence intra-hospitalière des hernies diaphragmatiques, types de hernies rencontrées, malformations associées, investigations demandées, voies d´abord, présence d´un sac herniaire, taille du défect et complications). La saisie et le stockage des données a été réalisée grâce au logiciel Microsoft Office Excel 2010 et l´analyse avec Epi Info. Les résultats seront présentés sous forme de texte et de tableaux. Les données manquantes ont été exclues lors de l´analyse de la variable concernée.

## Résultats

**Participants:** nous avons colligé un total de 20 patients dont huit ont été exclus: quatre pour décès préopératoire, deux pour dossiers inexploitables, un pour hernie diaphragmatique post-traumatique et un autre pour hernie paraœsophagienne. Au total, 12 patients ont été inclus dans notre étude.

**Données descriptives:** nous avons colligé 12 dossiers de patients qui répondaient à nos critères de sélection soit une fréquence moyenne annuelle de 1,2 cas par an. L´âge moyen de nos patients au moment du diagnostic était de 8,9 mois avec des extrêmes de un jour et 48 mois. Les nouveau-nés étaient au nombre de quatre. Notre série était composée de sept garçons et cinq filles.

L´âge maternel variait entre 18 et 42 ans. Une échographie obstétricale était réalisée chez six mères. Le lieu de l´accouchement était précisé chez huit patients dont sept dans une structure sanitaire périphérique et un à domicile. Parmi ces sept patients, les six étaient des accouchements à terme par voie basse et le septième une césarienne. Le score d´apgar était précisé chez six patients. Ce score variait entre 8/10 et 10/10. Le poids de naissance était rapporté chez six patients avec des extrêmes de 2720g et 4000g. Huit patients étaient référés des structures sanitaires périphériques. Les motifs de référence étaient une dyspnée chez cinq enfants, une détresse respiratoire chez deux enfants et des douleurs abdominales associées à des vomissements et un trouble du transit chez un enfant.

### Principaux résultats

**Malformations associées:** sept hernies postéro-latérales (HPL) étaient situées à gauche et les trois autres à droite. Les hernies rétro-costo-xiphoïdiennes (HRCX) siégeaient l´une à gauche et l´autre à droite. Cinq patients avaient des malformations ou anomalie chromosomique associées à la HDC dont une trisomie 21, une cryptorchidie, deux communications interauriculaires (CIA) et une hernie ombilicale à collet moyen. Les malformations digestives associées étaient représentées par des anomalies de rotation et de fixation, retrouvés chez tous les cinq patients: deux volvulus gastriques intrathoraciques, un défaut d´accolement de l´estomac, une malrotation intestinale et deux défauts de fixation du côlon.

**Symptômes:** neuf patients ont manifesté leurs premiers symptômes avant l´âge d´un mois et trois ont vu apparaître leur symptomatologie après un mois de vie. Les principaux symptômes présentés par nos malades étaient respiratoires (83,3% des patients), au premier rang desquels la détresse respiratoire, présente chez 7 patients. Les signes digestifs étaient présents chez cinq patients (41,6%), au premier rang desquels des vomissements chez quatre enfants. L´examen physique était dominé par un tirage intercostal, présent chez 6 patients.

**Examens complémentaires:** huit patients ont été diagnostiqués de façon tardive après la période néonatale. Des trois nouveau-nés, un seul patient était diagnostiqué dès la naissance par une radiographie thoraco-abdominale réalisée pour détresse respiratoire néonatale immédiate, suivie d´une TDM thoraco-abdominale réalisée à J1 de vie.

La radiographie thoracique était pratiquée chez tous les enfants et était largement évocatrice du diagnostic. La présence de clartés digestives intrathoraciques représentait le signe sémiologique le plus évocateur et le plus fréquemment retrouvé chez tous les patients de notre série. L´échographie thoraco-abdominale a été réalisée chez un seul patient et avait montré la présence d´anses digestives intrathoraciques. Le Transit œso-gastro-duodénal (TOGD) réalisé chez un enfant avait confirmé le diagnostic en montrant un volvulus gastrique dans le sens mésentérico-axial sur HDC. La tomodensitométrie (TDM) thoraco-abdominale a été réalisée chez 5 patients et avait permis d´identifier les viscères herniés. L´échocardiographie doppler était réalisée chez 11 patients. L´examen était normal chez 7 patients. Elle a objectivé une hypertension artérielle pulmonaire (HTAP) chez deux patients dont l´un avec insuffisance tricuspidienne importante et une communication inter-auriculaire (CIA) chez deux patients.

**Traitement:** trois patients ont bénéficié de stabilisation préopératoire pendant 24 à 48 heures, dont deux avec du sildénafil. Tous les patients ont subi une laparotomie sous-costale comme voie d´abord. Dix patients sur douze avaient un sac herniaire et la taille du défect a été rapportée chez neuf patients, variant de trois à dix centimètres de diamètre (Tableau 1). Les organes herniés étaient le côlon, le grêle, suivi de l´estomac, le foie et la rate (Tableau 2). Le sac herniaire a été réséqué dans tous les cas où il était présent (dix). La cure s´est faite avec des points séparés chez 11 patients dont 10 en X et un en U. Un patient a bénéficié d´un surjet. Les fils utilisés étaient du polyester chez dix patients et du polyglicatine chez deux patients. Un drain thoracique a été mis en place chez un seul patient.

**Tableau 1:** répartition des patients selon la taille du défect de la hernie diaphragmatique congénitale, recrutés à l’HEAR entre 2010 et 2019 (N=12)

Taille du défect	Effectif	Pourcentage
<5 cm	3	25%
5-10 cm	6	50%
Non précisé	3	25%
Total	12	100%

**Tableau 2:** répartition des patients selon les viscères herniés identifiés en peropératoire lors de la période de recrutement allant de 2010 à 2019, à l’HEAR (N=12)

Viscères herniés	Effectif	Pourcentage
Côlon	10	83,3%
Grêle	9	75%
Estomac	5	41,7%
Foie	5	41,7%
Rate	5	41,7%

**Suites opératoires:** la reprise du transit a varié d´un à quatre jours, avec une moyenne de 2,8 jours. La durée d´hospitalisation a varié de 3 à 20 jours, avec une moyenne de 10,7 jours. Dix patents sur douze n´ont présenté aucune complication postopératoire. Un patient a développé un pneumothorax gauche dont l´évolution a été favorable après sept jours de drainage. Un patient polymalformé est décédé au J1 postopératoire à la suite d´une obstruction de sa sonde d´intubation. Notons que ce patient était porteur d´un poumon gauche très comprimé, une HTAP associée à une CIA et une insuffisance tricuspidienne importante. Il avait bénéficié stabilisation préopératoire au sildénafil.

## Discussion

Notre étude a trouvé une moyenne d´âge de 8,9 mois, avec une large majorité des hernies de Bochdalek. Les symptômes les plus fréquents étaient respiratoires, puis digestifs. La radiographie thoraco-abdominale a permis d´évoquer le diagnostic chez tous nos patients. Tous ont bénéficié d´une laparotomie et 50% des patients avaient un défect mesurant entre 5 et 10 cm. Les suites opératoires ont été simples chez 10 patients et un décès a été enregistré, concernant un patient polymalformé.

La hernie diaphragmatique congénitale est une pathologie rare, sa prévalence globale a été rapportée à 2,3 pour 10.000 naissances [1]. En Afrique, les études basées sur la population sont presque inexistantes. Cependant, des prévalences hospitalières annuelles variant de 0,9 à 5,4 ont été rapportées [4-6]. Notre prévalence hospitalière de 1,2 cas par an est comprise dans cet intervalle et rejoint donc les données de la littérature en milieu Africain.

L´âge de diagnostic de la HDC varie selon qu´il s´agit d´une présentation néonatale ou retardée. Dans les présentations précoces, le diagnostic est anténatal dans les milieux à haut revenu dans environ 73% et néonatal dans 22% [2]. Dans les présentations tardives, l´âge moyen du diagnostic varie de 5 à 30 mois [4,7-9]. Notre série a trouvé un âge moyen de 8,2 mois, ce qui est proche des valeurs rapportées par la littérature lors des présentations tardives. Cela pourrait se justifier par le fait que la large majorité de nos patients se sont présentés après la période néonatale. La HDC survient différemment dans les deux sexes, les garçons étant les plus touchés [2,3,10]. Notre série confirme également cette tendance. Le lieu idéal de l´accouchement d´un nouveau-né porteur d´une HDC est un centre tertiaire [11]. Aucun de nos patients n´est né dans une structure tertiaire. Cela n´est qu´une retombée de l´absence de diagnostic anténatal, conduisant à un accouchement en périphérie, comme pour la plupart des grossesses normales dans notre milieu.

La fréquence des malformations associées à la HDC varie selon la taille des échantillons. Dans des larges revues, elles surviennent dans 28,2 à 40%, alors que dans les échantillons modestes, elles atteignent jusqu´à 80% [2,3,12,13]. Notre série rapporte au moins une malformation associée dans 41,7% des cas, ce qui rejoint les données de la littérature. Les malformations cardiaques sont les plus fréquentes avec 11 à 15%, suivies des malformations de l´arbre urinaire et des membres dans 5,5% des cas [3,13,14]. En ce qui concerne nos patients, les malformations cardiaques ont représenté 16,7% et aucune malformation de l´arbre urinaire n´a été rapportée. Cependant, les anomalies de rotation et/ou de fixation étaient présents chez tous les cinq patients avec malformations associées. Ces anomalies sont réputées être très fréquentes dans les HDC, atteignant jusqu´à 75% [14]. Dans une autre série, la hernie ombilicale a été rapportée dans 12,5% [14], ce qui est proche de notre série, avec 8,3%. Les anomalies génétiques surviennent dans 10% des cas dont 27% sont syndromiques et 73% chromosomiques [1,15,16]. Parmi nos patients, seul un cas de trisomie 21 a été détecté comme anomalie chromosomique. Cela pourrait être liée à la rareté de la réalisation du caryotype vu les contraintes financières auxquelles nous faisons face dans les pays à faible revenu.

Parmi nos patients, 75% ont présenté une symptomatologie avant l´âge d´un mois, mais n´ont consulté que tardivement, en étant déjà des nourrissons. Ceci ouvre une voie réflexion sur le retard de consultation comme facteur de risque des présentations tardives en milieu à faible revenu. La présentation tardive, au-delà de la période néonatale, se caractérise par quatre éventualités: asymptomatique dans 10,5 à 11%, symptômes respiratoires dans 43 à 52,6%, symptômes digestifs dans 26,3 à 33% ou les deux 10,5 à 13% [8,9,17]. Les résultats de notre échantillon confirment cette tendance, avec des signes respiratoires dans 58,3% et ceux digestifs dans 41,6%. La radiographie est suffisante pour poser le diagnostic dans 40 à 98,5% des cas [7,18-20]. Tous les patients de notre série ont bénéficié d´une radiographie thoraco-abdominale laquelle a permis le d´évoquer le diagnostic. D´autres examens sont utiles, notamment l´échographie cardiaque qui permet de rechercher une hypertension pulmonaire, d´évaluer la fonction cardiaque et d´exclure les malformations cardiaques associées [11]. Dans notre série, 91,7% des patients en ont bénéficié. Cela a permis de détecter des anomalies chez quatre d´entre eux, notamment: deux hypertensions pulmonaires et deux communications interauriculaires (CIA). Le transit œso-gastro-duodénal permet de confirmer la topographie des hernies de Morgani si la radiographie n´est pas contributive. Il peut aussi être demandé pour différencier les malformations congénitales kystiques des poumons de la HDC. Devant une symptomatologie de volvulus gastrique, le TOGD peut être le premier examen demandé [11]. Cette dernière éventualité a motivé sa réalisation chez un de nos patients. L´échographie diaphragmatique ou la fluoroscopie peuvent être demandées pour différencier la HDC et de l´éventration diaphragmatique alors que la TDM et l´IRM sont rarement indiqués [11]. Dans notre série, 41,7% des patients ont bénéficié de cet examen.

La stabilisation a pour objectif d´assurer une bonne oxygénation, une baisse des pressions des voies aériennes et une pression artérielle normale pour l´âge [21]. Dans notre série, 25% des patients ont bénéficié d´une stabilisation préopératoire faite d´administration du sildénafil. Cette faible représentation est liée au fait que la majorité des patients de notre échantillon se sont présenté tardivement. Plusieurs voies d´abord sont possibles, mais la laparotomie est l´abord le plus utilisé [22]. Dans une large revue, le groupe d´étude sur la HDC a rapporté que la laparotomie était réalisée dans 91,4%, la thoracotomie dans 3,1%, la laparotomie combinée à la thoracotomie dans 1,4%, la laparoscopie dans 0,6% et la thoracoscopie dans 2,6% [22]. Dans notre série, tous les patients ont bénéficié d´une laparotomie. Cela serait lié aux préférences des opérateurs, vu que la coelioscopie, qui était encore récente dans notre service, ne présente pas beaucoup d´avantages comparativement à la laparotomie qui offre une bonne visualisation, elle permet le diagnostic et la correction des malrotations, l´inspection des viscères réduits et la résection du sac, s´il est présent [23].

La HDC survient généralement en postéro-latéral, c´est la hernie de Bochdalek. Celle-ci survient dans 70 à 75% des HDC, dont 85% se produisent à gauche, 13% à droite et 2% bilatéral. Les hernies antérieures, de Morgani, sont rencontrées dans 23 à 28% des HDC. Les lésions centrales sont objectivées dans 2 à 7% [11,18]. Nos résultats concordent avec ceux de la littérature. Aucune hernie centrale n'a été diagnostiquée. Les viscères herniés sont variables selon le côté de la lésion. L´estomac dans 40 à 63,2%, le côlon ou l´entièreté de l´intestin dans 57,9%, la rate ou le foie dans 36,8% et le rein dans 5,2% des cas peuvent faire hernie [7,9]. Cette tendance se rapproche de celle de notre échantillon avec une hernie du côlon dans 83,3%, celle du grêle dans 75% et celles de l´estomac, la rate et le foie dans 41,7% des cas.

Le sac est présent dans 7,6 à 20% des cas [8]. Dans notre série, il a été retrouvé dans 83,3% des cas. Cette différence pourrait s´expliquer par la faible taille de notre échantillon comparativement aux larges compilations des données de la littérature. La résection du sac est obligatoire pour prévenir un épanchement pleural [11]. Cela a été réalisé chez tous nos patients. Devant une lésion de petite taille, la suture se fait avec du fil non résorbable. Pour les larges défects, la réparation se fait soit avec un lambeau du muscle grand dorsal ou des muscles abdominaux, soit avec une prothèse biologique ou synthétique. La mise en place systématique d´un drain thoracique n´est pas recommandée car pouvant causer un barotraumatisme [11,18,23]. Tous nos patients ont bénéficié d´une cure au fil non résorbable, sans besoin de prothèse, ce qui rejoint les tendances de la littérature où dans les laparotomies, la réparation primaire (sans prothèse) se fait dans 51,9 à 100% des cas [7,17,22]. Un seul patient a bénéficié d´un drainage thoracique.

L´abord abdominal devrait être fermé sans tension. Si cela est impossible, à cause de l´hyperpression causée par la réintégration des viscères herniés, une prothèse est alors utilisée [11,21]. Dans notre série, tous les patients ont bénéficié d´une suture sans tension. Cela serait dû au fait que dans les présentations tardives, la cavité abdominale soit suffisamment large pour contenir les viscères réduits.

La durée d´hospitalisation dépend des malformations associées et de la survenue des complications postopératoires. Dans notre série, elle a varié de 3 à 20 jours, ce qui se trouve dans les valeurs rapportées les présentations tardives, où elle varie de 5 à 22 jours au décours des laparotomies, alors qu´en cas de laparoscopie, elle peut être réduite à 3 jours [4,24].

Les suites opératoires immédiates des HDC sont généralement simples. Les complications postopératoires précoces sont généralement l´infection du site opératoire, le saignement (en cas d´utilisation de l´ECMO), la récidive précoce (pour les hernies de grande taille réparées par coelioscopie), le chylothorax (dans les réparations utilisant une prothèse et celles utilisant l´ECMO) et le syndrome compartimental intraabdominal, l´épanchement pleural (fréquemment rencontrée mais nécessitant rarement un drainage) [23]. Ce dernier a été rencontré chez un de nos patients, dont l´évolution s´est bien faite sous drainage thoracique. Cependant, certaines séries rapportent l´absence de complication postopératoire immédiate [4,8,24].

La mortalité des HDC est influencée par plusieurs facteurs dont la taille de la lésion, le poids de naissance, les gaz du sang, l´hypertension pulmonaire, l´association aux anomalies chromosomiques et malformations cardiaques [12,23-25]. Certaines séries rapportent une survie proche de 90% [25]. Dans notre série, la survie après une semaine est de 91,7%, ce qui est proche des valeurs rapportées par une large revue évaluant la survie à une semaine après la chirurgie qui l´a calculée entre 69,3% et à 72,7% dans les formes isolées [3]. Cependant, les études basées sur la population rapportent une survie allant de 42 à 68%. Cette différence serait probablement liée à la mortalité cachée des HDC. Cette notion fait appel aux décès in-utero et à ceux qui surviennent avant l´admission en milieu hospitalier [11,25].

**Limites:** 1) La première limite est le fait que cette étude est rétrospective, cela implique des données manquantes, lesquelles auraient été intéressantes à analyser: timing de l´échographie anténatale, lieu d´accouchement et score d´Apgar; 2) la seconde limite est le fait que l´étude ne rapporte pas de suivi à long terme, lequel permettrait d´évaluer le devenir des patients, y compris la survenue des récidives et des complications de la chirurgie abdominale. Cependant, ces limites n´enlèvent rien à la qualité des renseignements fournis par cette étude.

## Conclusion

La hernie diaphragmatique congénitale est une pathologie rare. Son diagnostic anténatal est presque inexistant dans les pays à faible revenu et le chirurgien pédiatre de ces régions, le plus souvent, prend en charge les présentations tardives, pour lesquelles la radiographie permet largement d´évoquer le diagnostic. La prise en charge par laparotomie reste la plus bénéfique et les résultats postopératoires sont gratifiants. Cependant, des études sur les résultats à long terme sont nécessaires.

### Etat des connaissances sur le sujet

- La hernie diaphragmatique congénitale est une pathologie rare dont la présentation peut être précoce ou tardive;
- Le traitement est chirurgical et la survie dépend des malformations associées.

### Contribution de notre étude à la connaissance

- Un état des lieux sur la prise en charge des hernie diaphragmatiques en Afrique sub-Saharienne;
- La possibilité qu´en milieu à faible revenu, la présentation tardive soit davantage liée à un retard de consultation qu´à un retard du début de la symptomatologie;
- La survie à une semaine est semblable à celle des pays à haut revenu où l´arsenal thérapeutique est bien plus important que dans nos contrées.

## References

[1] Paoletti M, Raffler G, Gaffi MS, Antounians L, Lauriti G, Zani A (2020). Prevalence and risk factors for congenital diaphragmatic hernia: a global view. J Pediatr Surg.

[2] Wright JC, Budd JL, Field DJ, Draper ES (2011). Epidemiology and outcome of congenital diaphragmatic hernia: a 9-year experience. Paediatr Perinat Epidemiol.

[3] McGivern MR, Best KE, Rankin J, Wellesley D, Greenlees R, Addor MC (2015). Epidemiology of congenital diaphragmatic hernia in Europe: a register-based study. Arch Dis Child Fetal Neonatal Ed.

[4] Abdur-Rahman LO, Bamigbola KT, Adeoye PO, Akande HJ, Nasir AA, Obasa TO (2016). Late presentation of congenital diaphragmatic hernia in sub-Saharan Africa: a call for screening and prompt treatment. J Med Trop.

[5] Abubakar AM, Bello MA, Chinda JY, Danladi K, Umar IM (2011). Challenges in the management of early versus late presenting congenital diaphragmatic hernia in a poor resource setting. Afr J Paediatr Surg.

[6] Numanoglu A, Morrison C, Rode H (1998). Prediction of outcome in congenital diaphragmatic hernia. Pediatr Surg Int.

[7] Elhalaby EA, Abo Sikeena MH (2002). Delayed presentation of congenital diaphragmatic hernia. Pediatr Surg Int.

[8] Kitano Y, Lally KP, Lally PA, Congenital Diaphragmatic Hernia Study Group (2005). Late-presenting congenital diaphragmatic hernia. J Pediatr Surg.

[9] Cigdem MK, Onen A, Otcu S, Okur H (2007). Late presentation of bochdalek-type congenital diaphragmatic hernia in children: a 23-year experience at a single center. Surg Today.

[10] Tovar JA (2012). Congenital diaphragmatic hernia. Orphanet J Rare Dis.

[11] McHoney M, Lakhoo K (2020). Congenital diaphragmatic hernia and diaphragmatic eventration. Pediatric Surgery.

[12] Hosgor M, Karaca I, Karkiner A, Ucan B, Temir G, Erdag G (2004). Associated malformations in delayed presentation of congenital diaphragmatic hernia. J Pediatr Surg.

[13] Leeuwen L, Fitzgerald DA (2014). Congenital diaphragmatic hernia. J Paediatr Child Health.

[14] Graziano JN, Congenital Diaphragmatic Hernia Study Group (2005). Cardiac anomalies in patients with congenital diaphragmatic hernia and their prognosis: a report from the Congenital Diaphragmatic Hernia Study Group. J Pediatr Surg.

[15] Shanmugam H, Brunelli L, Botto LD, Krikov S, Feldkamp ML (2017). Epidemiology and prognosis of congenital diaphragmatic hernia: a population-based cohort study in Utah. Birth Defects Res.

[16] Samangaya RA, Choudhri S, Murphy F, Zaidi T, Gillham JC, Morabito A (2012). Outcomes of congenital diaphragmatic hernia: a 12-year experience. Prenat Diagn.

[17] Baerg J, Kanthimathinathan V, Gollin G (2012). Late-presenting congenital diaphragmatic hernia: diagnostic pitfalls and outcome. Hernia.

[18] Puri P (2019). Congenital diaphragmatic hernia and eventration. Pediatric Surgery.

[19] Newman BM, Afshani E, Karp MP, Jewett TC, Cooney DR (1986). Presentation of congenital diaphragmatic hernia past the neonatal period. Arch Surg.

[20] Carter RE, waterston DJ, Aberdeen E (1962). Hernia and eventration of the diaphragm in childhood. Lancet.

[21] de Buys Roessingh AS, Dinh-Xuan AT (2009). Congenital diaphragmatic hernia: current status and review of the literature. Eur J Pediatr.

[22] Tsao K, Lally PA, Lally KP, Congenital Diaphragmatic Hernia Study Group (2011). Minimally invasive repair of congenital diaphragmatic hernia. J Pediatr Surg.

[23] Montalva L, Zani A (2021). Congenital diaphragmatic hernia. Pediatric Surgery.

[24] Kim DJ, Chung JH (2013). Late-presenting congenital diaphragmatic hernia in children: the experience of single institution in Korea. Yonsei Med J.

[25] Downard CD, Jaksic T, Garza JJ, Dzakovic A, Nemes L, Jennings RW (2003). Analysis of an improved survival rate for congenital diaphragmatic hernia. J Pediatr Surg.

